# *Polygonum minus* essential oil modulates cisplatin-induced hepatotoxicity through inflammatory and apoptotic pathways

**DOI:** 10.17179/excli2020-2355

**Published:** 2020-09-09

**Authors:** Norhashima Abd Rashid, Farida Hussan, Asmah Hamid, Nurul Raudzah Adib Ridzuan, Syarifah Aisyah Syed Abd Halim, Nahdia Afiifah Abdul Jalil, Nor Haliza Mohamad Najib, Seong Lin Teoh, Siti Balkis Budin

**Affiliations:** 1 Center for Diagnostic, Therapeutic and Investigative Studies, Faculty of Health Sciences, Universiti Kebangsaan Malaysia, Kuala Lumpur, Malaysia; 2 Human Biology Department, School of Medicine, International Medical University, Bukit Jalil, Kuala Lumpur, Malaysia; 3 Centre for Toxicology and Health Risk Studies, Faculty of Health Sciences, Universiti Kebangsaan Malaysia, Kuala Lumpur, Malaysia; 4 Department of Anatomy, Faculty of Medicine, Universiti Teknologi MARA, Selangor, Malaysia; 5 Department of Anatomy, Faculty of Medicine, Universiti Kebangsaan Malaysia Medical Centre, Kuala Lumpur, Malaysia

**Keywords:** apoptosis, cisplatin, hepatotoxicity, inflammation, oxidative stress, Polygonum minus essential oil

## Abstract

Oxidative stress, inflammation and apoptosis are thought as primary mediators of cisplatin-induced hepatotoxicity. The objective of this study was to determine the protective effect of *Polygonum minus* essential oil in cisplatin-induced hepatotoxicity. A total of forty-two male rats were randomly divided into seven groups: control, cisplatin, β-caryophyllene 150 mg/kg (BCP), PmEO 100 mg/kg + cisplatin (PmEO100CP), PmEO 200 mg/kg + cisplatin (PmEO200CP), PmEO 400 mg/kg + cisplatin (PmEO400CP) and PmEO 400 mg/kg (PmEO400). Rats in the BCP, PmEO100CP, PmEO200CP, PmEO400CP and PmEO400 group received respective treatment orally for 14 consecutive days prior to cisplatin injection. All animals except for those in the control group and PmEO400 were administered with a single dose of cisplatin (10 mg/kg) intraperitoneally on day 15 and all animals were sacrificed on day 18. PmEO100CP pretreatment protected against cisplatin-induced hepatotoxicity by decreasing CYP2E1 and indicators of oxidative stress including malondialdehyde, 8-OHdG and protein carbonyl which was accompanied by increased antioxidant status (glutathione, glutathione peroxidase, superoxide dismutase and catalase) as compared to cisplatin group. PmEO100CP pretreatment also modulated changes in liver inflammatory markers (TNF-α, IL-1α, IL-1β, IL-6 and IL-10). PmEO100CP administration also notably reduced cisplatin-induced apoptosis significantly as compared to cisplatin group. In conclusion, our results suggested that *P. minus* essential oil at a dose of 100 mg/kg may protect against cisplatin-induced hepatotoxicity possibly via inhibition of oxidative stress, inflammation and apoptosis.

## Introduction

Cancer has emerged as one of the major causes of death worldwide. Cisplatin is an effective chemotherapeutic drug used to treat various types of cancers such as cancers of testis, lung, colon, cervix and ovary (Ko et al., 2014[50]); however its usage may also cause various side effects including hepatotoxicity. Cisplatin tends to accumulate in liver (Gong et al., 2015[35]; Lebwohl and Canetta 1998[52]) and caused hepatotoxicity in cancer patients after a high dose of treatment (40 - 100 mg/m^2^) (Al-Malki and Sayed, 2014[7]; Cersosimo, 1993[19]). Oxidative stress plays an important role in cisplatin-induced hepatotoxicity. Administration of cisplatin especially at higher dose results in hepatotoxicity via formation and activity of reactive oxygen species (ROS) (Cichoż-Lach and Michalak, 2014[23]; Cüre et al., 2016[25]). Excessive ROS can stimulate oxidative damage through oxidation of macromolecules such as protein, lipid and DNA. Dysregulation of pro-inflammatory cytokines and unregulated apoptosis also play an important role in cisplatin-induced hepatotoxicity (El-Shitany and Eid, 2017[32]).

In recent years, researchers are increasingly keen on herbal products for use in daily health care products or as food supplements. Numerous herbs including *Psidium guajava *(Alias et al., 2015[6]), *Hibiscus sabdariffa *(Akhtar Husin et al., 2017[5]) and *Zingiber zerumbet *(Hamid et al., 2018[36]) were reported to exhibit hepatoprotective effects and high antioxidant activity. Given that current evidence suggesting that cisplatin-induced hepatotoxicity is mediated by oxidative stress (Karadeniz et al., 2011[44]; Palipoch et al., 2014[64]); natural products with antioxidant activity could be used as an alternative treatment to overcome this problem. 

β-Caryophyllene is a volatile bicyclic sesquiterpene compound which is abundantly discovered in essential oils of many plants such as cloves, oregano, cinnamon, rosemary and Copaiba oil (De Almeida Borges et al., 2013[29]; Khani and Heydarian, 2014[47]). It has shown to exhibit antioxidant, anti-inflammatory and anti-cancer activities. A previous study revealed that β-caryophyllene has therapeutic potential as a hepatoprotective effect in carbon tetrachloride-induced fibrosis (Calleja et al., 2013[17]). Cho and colleagues (2015[22]) also demonstrated β-caryophyllene has protective effects against liver failure induced by D-galatosamine and LPS in mice. In addition, β-Caryophyllene also effectively improved cisplatin-induced nephrotoxicity and decreased the pro-inflammatory cytokine expression in rats (Horváth et al., 2012[39]). Interestingly, *P. minus* essential oil was found to have high concentration of β-caryophyllene (Ahmad et al., 2014[2]).

Previous studies have shown that essential oil has hepatoprotective effects in various animal models (Bouzenna et al., 2016[14]; Jiang et al., 2019[43]; Morales-López et al., 2017[58]; Özbek et al., 2016[62]; Selmi et al., 2015[73]). *P. minus* contains various medicinal values and yields high levels of essential oil (72.54 %) mainly of aliphatic aldehydes, terpenoids and organic acids (Baharum et al., 2010[10]). Previous researchers also identified monoterpenes and sesquiterpenes in *P. minus* essential oil including ɑ-humulene, ɑ-curcumene, β-caryophyllene, δ-cadinene, farnesol and nerolidol (Baharum et al., 2010[10]; Rusdi et al., 2016[71]). To the best of our knowledge, hepatoprotective effects of *P. minus* essential oil in cisplatin-induced hepatotoxicity model is unknown although it yields high levels of essential oil with proven antioxidant activities. The purpose of this study was therefore to investigate the possible protective effects of *P. minus* essential oil on cisplatin-induced hepatotoxicity in adult male rats with emphasis on oxidative stress, inflammation and apoptosis.

## Materials and Methods

### Essential oil extraction

*P. minus *leaves were collected from Haji Hussin Markom Sdn. Bhd farm near Rawang, Selangor, Malaysia (3.3453364° N 101.593589° E). *P. minus* was then authenticated at the Institutional Herbarium Department, Faculty of Science and Technology, Universiti Kebangsaan Malaysia (No. UKMB40332). Fresh entire leaves (10 kg) of *P. minus* were subjected to hydrodistillation with 40 L of distilled water for 8 hours using a Clevenger-type apparatus as described previously (Ahmad et al., 2014[2]). Essential oil was collected over distilled water, separated, and dried over nitrogen gas prior to storage in dark at 4 °C. 

### Gas chromatography-mass spectrometry (GC-MS) analysis

*P. minus* essential oil compounds were identified using GC-MS analysis (Ahmad et al., 2014[2]) using the Clarus 600 GC-MS system (PerkinElmer, USA). Volatile compounds were separated using a 30 m x 0.25 mm x 0.25 µm Elite-5MS column. The injector port was heated to 250 °C with helium as carrier gas at a constant flow of 1 mL·min^-1^. All mass spectra were acquired in the electron impact mode (EI). The MS parameters were as follows: EI mode, an ionization voltage of 70 eV, an ion source temperature of 200 °C and a scan range of 40 - 600 Da. The peaks were tentatively identified based on a library search using NIST and Wiley Registry 8 Edition (Ahmad et al., 2014[2]).

### Ethics statement

This study was carried out in strict accordance with the recommendations approved by the Universiti Kebangsaan Malaysia Animal Ethical Committee (Protocol Number: FP/ANAT/2014/FAIZAH/26-NOV./632-NOV.-2014-SEPT.-2017). Animals were anesthetized using ketamine/xylazine, and all efforts were made to prevent misconduct in animal handling.

### Animals

Forty-two adult male Sprague Dawley rats were obtained from Laboratory Resource Unit, Faculty of Medicine, Universiti Kebangsaan Malaysia (UKM). Rats (180 - 200 g) were kept in polycarbonate cages with good ventilation in animal house under standard conditions (12/12 light/dark cycle at 21 ± 2 °C and 50 - 70 % humidity). All animals were acclimatized to the laboratory environment for 7 days before treatment. Standard rodent pellets and tap water were provided *ad libitum* throughout the experimental period. 

### Experimental design

A total of forty-two male Sprague Dawley rats were randomly divided into seven groups: control, cisplatin, β-caryophyllene 150 mg/kg (BCP), *P. minus* essential oil (PmEO); PmEO 100 mg/kg + cisplatin (PmEO100CP), PmEO 200 mg/kg + cisplatin (PmEO200CP), PmEO 400 mg/kg + cisplatin (PmEO400CP) and PmEO 400 mg/kg (PmEO400). Rats in the BCP, PmEO100CP, PmEO200CP, PmEO400CP and PmEO400 group received respective treatment orally for 14 consecutive days (Abd Rashid et al., 2019[1]; Ebada, 2018[31]). Doses of *P. minus* essential oil were chosen according to Ebada (2018[31]) as well as Rojas-Armas and colleagues (2019[70]). Meanwhile, the dose of β-caryophyllene was based on Horváth and colleagues (2012[39]). β-Caryophyllene served as positive control. All animals except for those in the control group and PmEO400 group were administered with a single dose of cisplatin (10 mg/kg) intraperitoneally (Bishr et al., 2018[13]) on day 15, left untreated for three days and were sacrificed humanely on day 18 (Palipoch and Punsawad 2013[63]). Blood samples were collected via cardiac puncture, whereas liver was excised for further analysis. Supplementary Figure 1 in the supplementary data shows the flowchart of the experimental design.

### Analysis of serum biochemistry

The obtained sera were kept at -80 °C and used within 48 hours for assaying liver bio- markers; Alanine aminotransferase (ALT), Aspartate Aminotranferase (AST), Alkaline Phosphatase (ALP) and total bilirubin using an automated clinical analyzer (BioLis 24i Premium, Tokyo, Japan).

### Cytochrome P450 2E1 (CYP2E1) and liver apoptosis gene expression by quantitativepolymerase chain reaction 

Total RNA was isolated from the liver tissue using an RNeasy Plus Minikit (cat. n. 74136; Qiagen, Germany). The method was performed according to the manufacturer's guideline. RNA content and purity were measured using Nanodrop (DeNovix DS-11+ Spectrophotometer, DE USA). A total of 5 µg of total RNA was used for cDNA synthesis using Quantinova^TM^ Reverse Transcription Kit (cat. n. 205413; Qiagen, Germany). For qPCR analysis, the cDNA samples were run in triplicate and β-actin was used as a reference gene. Each PCR amplification cycle includes non-template controls that consist of all reagents except for cDNA. qPCR reactions were performed using Quantinova SYBR^®^ Green RT-PCR Kit (cat. n. 208154; Qiagen, Germany) and was conducted using the CFX96 Touch^TM ^Real-Time PCR Detection System (BioRad, USA). After the qPCR reaction, the quantitation cycle (Cq) was determined for each sample from the amplification plot. ΔΔCq value was calculated by subtraction of the β-actin Cq from each sample Cq and used for data analysis. PCR primers for *p53*, *Caspase* (*8*, *9* and *3*), *Bcl-2*, *Bax*, *APAF-1* and *CYP2E1* were synthesized by Matrioux Sdn. Bhd. (Malaysia). Primers were designed using the Primer-Blast program from NCBI. PCR primer sequences (Table 1(Tab. 1)) were obtained in and verified using BLAST to ensure for specificity for the particular gene. 

### Western Blot for cytochrome c analysis

Liver tissue homogenates were prepared for Western blotting as mentioned by Omar and colleagues (2016[60]). Briefly, protein concentration in the cytosolic fraction was determined using protein assay kit (Bio Rad, USA). According to Chen and colleagues (2000[21]), the release of cytochrome c from mitochondria into cytosol is the critical early event leading to apoptosis. Protein samples were then separated by SDS-PAGE (10 µg per lane) and transferred onto a PVDF membrane. The membrane was blocked using 5 % (w/v) of bovine serum albumin (BSA) in Tris-buffered saline with Tween-20®, incubated for overnight at 4 °C with primary antibody (Cytochrome C (D18C7) Rabbit, cat. n. 11940; Cell Signalling, USA) with dilution factor 1:1000, rinsed and then incubated with 1:2000 secondary antibody (Anti-rabbit IgG, HRP-linked antibody; cat. n. 7074; Cell Signalling, USA) for 1 hour at room temperature before the detection using Clarity Western ECL Substrate (Bio Rad, USA). β-Actin (β-Actin (D6A8) Rabbit; cat. n. 8457; Cell Signalling, USA) was used as a loading control. Band density in intermediately exposed films was quantified using Image Lab^TM^ 6.0 (Bio Rad, USA).

### Analysis of liver oxidative stress

A portion of rat liver was homogenized in 10 % phosphate buffered saline (0.05M, pH 7.4) and was centrifuged at 15,000 x g for 15 min at 4 °C. The supernatant was collected and used for measurement of glutathione (GSH), superoxide dismutase (SOD), catalase, glutathione peroxidase (GPx), malondialdehyde (MDA) (Cayman Chemical, USA), protein carbonyl and 8-hydroxyde-oxyguanosine (8-OHdG). Antioxidant markers, GSH (cat. n. 703002), SOD (cat. n. 706002), catalase (cat. n. 707002), GPx (cat. n. 703102), MDA (cat. n. 1009055) (Cayman Chemical, USA) and 8-OHdG (cat. n. E-EL-0028; Elabscience, China) were all measured using commercial ELISA kits according to the manufacturer's guidelines.

Protein oxidation was measured with the level of protein carbonyl by using methods from Levine et al. (1990[53]). The determination of the protein carbonyl was based on the reaction with 2, 4-Dinitrophenylhydrazine forming a stable dinitrophenylhydrazone product that can be measured using a spectrophoto-meter at 370 nm.

### Assay of TNF-α, IL-1 (α and β), IL-6, IL-10

Level of cytokines in liver homogenates was measured using an available Multiplex ELISA kit (cat. n. RECYMAG65K27PMX; Milliplex^®^_MAP_ Rat Cytokine / Chemokine Magnetic Bead Panel; Merck. Massachusetts, USA) according to the manufacturer's instruction. 

### Statistical analysis

All values are expressed as mean ± SEM. Statistical analyses were performed using Statistical Package for the Social Sciences version 23 (SPSS Inc, IL, USA). Data with homogeneous intra-group variances were subjected to one-way analysis of variance (ANOVA) followed by *post-hoc* Tukey's test. *P* value of 0.05 or less was considered significant.

## Results

### Essential oil compound of P. minus leaves

The main volatile compounds found in *P. minus* essential oil were aldehydes, alcohols, sesquiterpenes as well as organic acids as shown in Supplementary Figure 2 and Table 2(Tab. 2) (References in Table 2: Ahmad et al., 2014[2]; Abd Rashid et al., 2019[1]). Dodecanal (39.4 %) and decanal (10.4 %) were the most dominant compounds identified in *P. minus* essential oil. Sesquiterpene found in the *P. minus* essential oil includes 8.61 % cyclolongifolene oxide, 3.3 % α-caryophellene, 1.13 % α-bergamotene, 0.72 % seychellene, 0.63 % β-caryophellene, 0.58 % velencene and 0.56 % isolongifolol.

### Effect of P. minus essential oil on liver function in cisplatin-induced hepatotoxicity rats 

ALT, AST, ALP and total bilirubin levels were all significantly increased in the cisplatin group when compared to the control group at p<0.05 (Table 3(Tab. 3)). Conversely, a significant reduction was also observed for ALT, AST and ALP and total bilirubin in PmEO100CP and PmEO200CP groups compared to the cisplatin group at p<0.05. However, the result showed no significant difference between the PmEO400CP group and the cisplatin group. For all measured parameters, PmEO400CP group showed a significant increase compared to all treatment groups at p<0.05. The supplementation of 400 mg/kg of *P. minus* essential oil alone showed significant increase in ALP and total bilirubin compared to the control group at p<0.05. Meanwhile, BCP group showed significantly improved ALT, AST, ALP and total bilirubin compared to the cisplatin group at p<0.05. 

### Effect of P. minus essential oil on CYP2E1 gene expression in the liver of cisplatin-induced hepatotoxicity rats 

**Figure 1:**
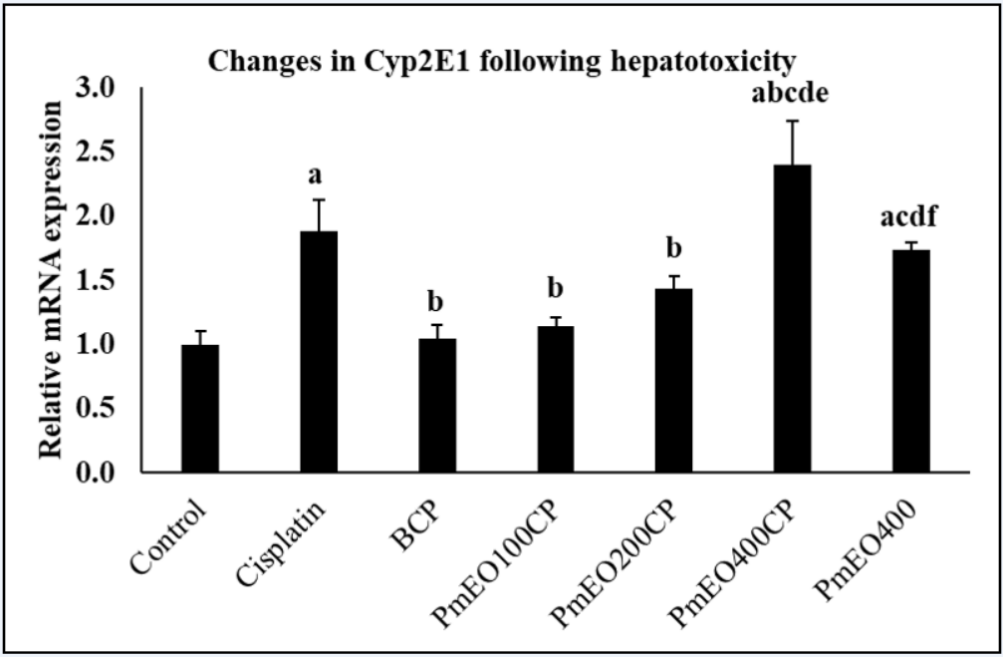
Effect of P. minus essential oil on CYP2E1 gene expression in the liver of cisplatin-induced hepatotoxicity rats. PmEO100CP and BCP groups have significantly lower expression of CYP2E1 compared to cisplatin group. In contrast, the expression of CYP2E1 was significantly higher in the PmEO400CP group. Values are mean ± SEM (n = 6). ^a^Significantly different from control group at p<0.05; ^b^Significantly different from cisplatin group p<0.05. ^c^Significantly different from BCP group at p<0.05. ^d^Significantly different from PmEO100CP group at p<0.05. ^e^Significantly different from PmEO200CP group at p<0.05. ^f^Significantly different from PmEO400CP group at p<0.05.

Figure 1(Fig. 1) shows mRNA expression level of CYP2E1 gene was significantly increased in the cisplatin group compared to the control group at p<0.05. Meanwhile, PmEO100CP and PmEO200CP has significantly lower CYP2E1 gene expression than cisplatin group and PmEO400CP at p<0.05. BCP group also showed significantly decreased CYP2E1 gene expression compared to the cisplatin group at p<0.05. Supplementary Table 1 shows mean and SEM of CYP2E1 expression level in rats.

### Effect of P. minus essential oil on antioxidant and oxidative stress biomarkers in the liver of cisplatin-induced hepatotoxicity rats 

Following cisplatin administration, level of GSH and activities of GPx, CAT and SOD decreased significantly corresponding to the significant increase in MDA, protein carbonyl and 8-OHdG level (all p<0.05 vs. control) (Table 4(Tab. 4)). The PmEO100CP group showed significantly improved GSH, GPx, CAT and SOD status whilst MDA, protein carbonyl and 8-OHdG were significantly lowered compared to the cisplatin group at p<0.05. PmEO100CP showed a better antioxidant status significantly compared with the PmEO200CP and PmEO400CP groups at p<0.05. Meanwhile, the BCP group showed significant improvement in all measured biomarkers compared to the cisplatin group at p<0.05.

### Effect of P. minus essential oil on inflammatory markers in the liver of cisplatin-induced hepatotoxicity rats

**Figure 2:**
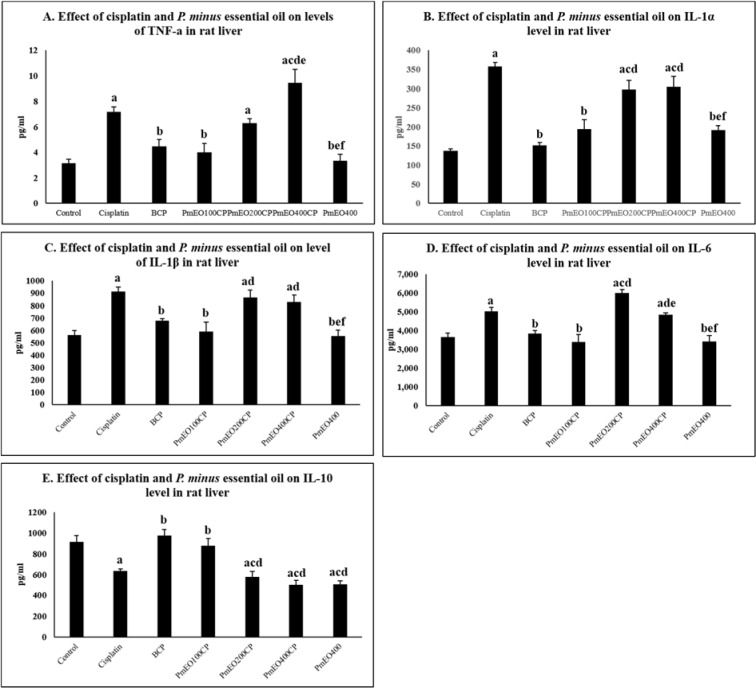
Effect of *P. minus* essential oil on inflammatory markers in the liver of cisplatin-induced hepatotoxicity rats. The levels of the inflammatory markers have been restored to the normal level in PmEO100CP and BCP groups. In contrast, this finding was not observed in PmEO200CP and PmEO400CP groups. Values are mean ± SEM (n=6). ^a^Significantly different from control group at p<0.05; ^b^Significantly different from cisplatin group at p<0.05. ^c^Significantly different from BCP group at p<0.05. ^d^Significantly different from PmEO100CP group at p<0.05. ^e^Significantly different from PmEO200CP group at p<0.05. ^f^Significantly different from PmEO400CP group at p<0.05.

Level of TNF-α, IL-1β and IL-6 was significantly increased in the cisplatin group compared to the control group at p<0.05 as shown in Figure 2(Fig. 2). Contrarily, IL-10 level was significantly decreased in the cisplatin group compared to the control group at p<0.05. The levels of TNF-α, IL-1β, IL-6 and IL-10 in the PmEO100CP group were all significantly corrected compared to the cisplatin group at p<0.05. Interestingly, this reduction was significant compared to the PmEO200CP and PmEO400CP groups at p<0.05. Meanwhile, TNF-α, IL-1β and IL-6 decreased significantly while IL-10 increased significantly in the BCP group compared to the cisplatin group at p<0.05. Supplementary Table 2 shows mean and SEM of the effect of cisplatin and *P. minus* essential oil on inflammation markers.

### Effect of P. minus essential oil on apoptosis gene expression in the liver of cisplatin-induced hepatotoxicity rats 

**Figure 3:**
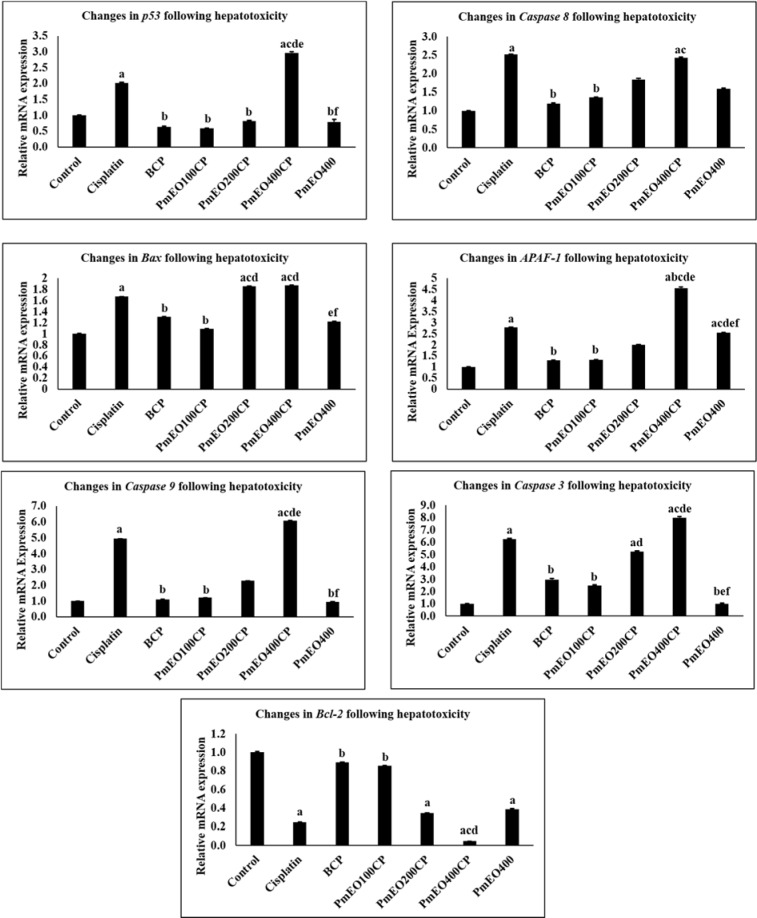
Effect of *P. minus* essential oil on apoptosis gene expression in the liver of cisplatin-induced hepatotoxicity rats. The expression of *p53*, *caspase 8*, *Bax*, *Apaf-1* and *caspase 3* in PmEO100CP and BCP groups were significantly lower compared to cisplatin group, however, the expression of *Bcl-2* showed significantly higher values. However, PmEO400CP groups showed contrary findings from PmEO100CP and BCP groups. Values are mean ± SEM (n = 6). ^a^Significantly different from control group at p<0.05; ^b^Significantly different from cisplatin group at p<0.05. ^c^Significantly different from BCP group at p<0.05. ^d^Significantly different from PmEO100CP group at p<0.05. ^e^Significantly different from PmEO200CP group at p<0.05. ^f^Significantly different from PmEO400CP group at p<0.05.

mRNA expression of p53, caspase 8, Bax, caspase 3, Bcl-2 in rat liver treated with cisplatin and *P. minus* essential oil is shown in Figure 3(Fig. 3). The mRNA expression of all the genes except for Bcl2, increased significantly in the cisplatin and PmEO400CP groups compared to the control group p<0.05. However, the expression of these genes was significantly decreased in PmEO100CP and BCP groups compared to the cisplatin group at p<0.05. In contrast, expression of Bcl-2 (anti-apoptotic gene) was significantly lowered in cisplatin, PmEO200CP, PmEO400CP and PmEO400 compared to the control group. Conversely, PmEO100CP showed a significant increase in Bcl2 gene expression at p<0.05. Supplementary Table 3 shows mean and SEM of the effect of cisplatin and *P. minus* essential oil on expression of genes related to apoptosis.

### Effect of P. minus essential oil on cytochrome c protein expression in the liver of cisplatin-induced hepatotoxicity rats 

**Figure 4:**
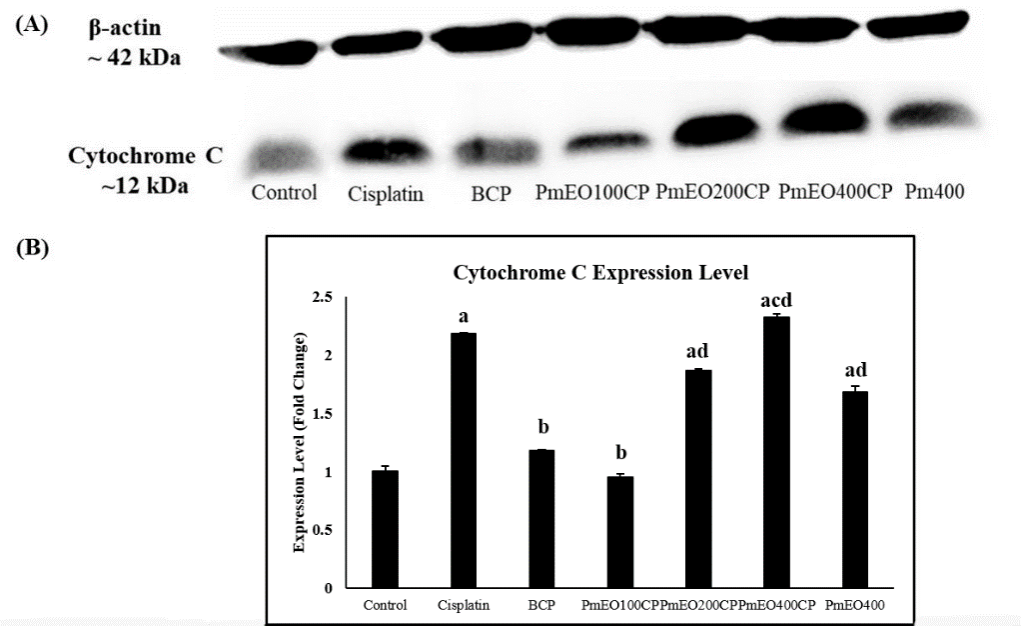
Effect of *P. minus* essential oil on cytochrome c protein expression in the liver of cisplatin-induced hepatotoxicity rats. (A) Western blot analysis showed the band of the cytochrome c protein expression was less thicken and less clearer in the PmEO100CP and BCP groups compared to cisplatin group. However, the band was thick and clear in PmEO200CP and PmEO400CP. (B) Quantitated immunoblotted proteins by using densitometry showed the expression of cytochrome c protein in PmEO100CP and BCP groups was normalized to the control level. However, PmEO200CP and PmEO400CP groups showed contrary findings. Column, mean; bars, ± SEM (n=3 independent experiments). ^a^Significantly different from control group at p<0.05. ^b^Significantly different from cisplatin group at p<0.05. ^c^Significantly different from BCP group at p<0.05. ^d^Significantly different from PmEO100CP group at p<0.05.

As shown in Figure 4A(Fig. 4), Western Blot analysis of cytochrome c protein expression band was a strong single band with molecular mass of ~12 kDa in the cytosolic fraction in the liver of cisplatin, PmEO200CP and PmEO400CP-treated rats. However, the cytochrome c band in control, BCP and PmEO100CP-treated rats are less clear and weaker compared to PmEO200CP and PmEO400CP. The amount of immunoblotted proteins was quantitated using densitometry. Figure 4B(Fig. 4) shows that cytochrome c expression level was significantly increased in cisplatin, PmEO200CP, PmEO400CP and PmEO400 compared to control group. However, the expression level of cytochrome c was significantly decreased in BCP and PmEO100CP compared to cisplatin group. The expression level of cytochrome c in PmEO200CP, PmEO400CP and PmEO400 groups was significantly increased compared to PmEO100CP group. Supplementary Table 4 shows mean and SEM of the effect of cisplatin and *P. minus* essential oil in cytochrome c protein expression.

## Discussion

The present study investigated the potentials of *P. minus* essential oil against cisplatin-induced hepatotoxicity. Cisplatin-induced hepatotoxicity occurs through the production of reactive oxygen species which depends on the concentration of platinum compound generated during chemotherapy (Brozovic et al., 2010[16]). Cisplatin causes hepatotoxicity by triggering oxidative stress and inflammation that further leads to apoptosis in liver (Bentli et al., 2013[11]; Omar et al., 2016[60]). This study was carried out using 10 mg/kg/body weight of cisplatin to produce hepatotoxicity corresponding to the dose of cisplatin currently used in the clinical practice (70 mg/m^2^) (Nair and Jacob, 2016[59]). 

The major volatile compound in *P. minus* essential oil are aldehydes, terpenoids and organic acids. Aldehydes, such as decanal and dodecanal while cyclolongifolene oxide, α-caryophyllene, β-caryophyllene, seychellene, α-bergamotene, valencene and isolongifolol are the main sesquiterpenes found in *P. minus* essential oil. These findings were similar with Rusdi and colleagues (2016[71]) as well as Ahmad and colleagues (2014[2]). α-Caryophyllene and β-caryophyllene were found to reduce the anti-inflammatory mediators in rats (Rogerio et al., 2009[69]). Besides that, β-caryophyllene was also found to improve cisplatin-induced nephrotoxicity in a cannabinoid 2 receptor-dependent manner (Calleja et al., 2013[17]). Seychellene, α-bergamotene and valencene were found to have high antioxidant, anti-inflammatory and also anti-apoptotic properties against chemical-induced toxicity in rats (Huang et al., 2018[40]; Su et al., 2012[77]; Tsai et al., 2010[81]; Tsoyi et al., 2011[82]).

To the best of our knowledge there is no previous study using either *in vitro *or* in vivo* research models to identify the beneficial effects of *P. minus* essential oil. However, there were studies on *P. minus* using aqueous, methanolic and ethanolic extract which showed no toxic effect at as much as 2000 mg/kg dose. Ebada (2018[31]) has shown that chamomile essential oil at a dose of 250 mg/kg and cumin essential oil at a dose of 400 mg/kg increased the serum transaminase and serum bilirubin in acetaminophen-induced hepatotoxicity. In addition, Rojas-Armas and colleagues (2019[70]) demonstrated that in a subacute toxicity study, a dose of 100 mg/kg *Thymus vulgaris* essential oil did not cause a significant difference in serum transaminase and total bilirubin level compared to the control group. Therefore, in order to determine the effective dose of *P. minus* essential oil, doses of 100, 200 and 400 mg/kg were chosen based on Ebada (2018[31]), as well as Rojas-Armas and colleagues (2019[70]). 

Cisplatin-induced hepatotoxicity is characterized by an increase in serum transaminases, and less frequently by the average increase in serum ALP and total bilirubin. The serum levels of ALT and AST act as oblique assessment of liver condition because these enzymes are normally found in the cytoplasm and are only secreted into the circulation after hepatic damage (Contreras-Zentella and Hernández-Muñoz, 2016[24]). In the present study, the capability of *P. minus* essential oil in protecting against cisplatin-induced hepatotoxicity was observed from a lowered level of liver damage biomarker at the dose of 100 and 200 mg/kg. This observation might be attributed to the protective effect of *P. minus* essential oil in reducing cell damage that may occur due to increased lipid and protein oxidation especially at the phospholipid bilayer membrane (Itri et al., 2014[42]). 

However, the result obtained from PmEO400CP showed a contrary finding compared to the PmEO100CP group which demonstrated the increased levels of liver enzymes and total bilirubin indicate the occurrence of liver injury. At higher concentration, *P. minus* essential oil contains high concentration of aldehyde. Aldehyde and acetone are much alike in many of their reactions owing to the presence of carbonyl functional groups (Ouellette and Rawn, 2014[61]). In line with the previous study a combination of acetone and cisplatin in rats accelerate the generation of ROS, reducing antioxidant status causing cellular injury and oxidative stress (Lu and Cederbaum, 2006[54]). Our findings also supported Tisserand and Young (2014[79]), stating that the action of some oil constituents depends on the dosage. They found that at lower concentration of clove essential oil protects human oral mucous membrane cells from the attack of ROS, however, at higher concentration, it causes cytotoxic effect.

Cytochrome P450 2E1 (CYP2E1) majorly expressed in the liver, enhances the formation of superoxide anion and hydrogen peroxide which finally produce powerful oxidants such as hydroxyl radical (Lu and Cederbaum, 2008[55]). Interaction of cisplatin with CYP2E1 promotes the production of ROS and leads to the enhancement of oxidative stress (Lu and Cederbaum, 2006[54]). In this study, supplementation of *P. minus* essential oil at a dose of 100 mg/kg in cisplatin treated rats inhibited the *CYP2E1* gene expression and thus it is likely to protect the liver from cisplatin toxicity. The protective effects exerted by *P. minus* essential oil could be related to the presence of α-caryophyllene and β-caryophyllene, which have been reported to down-regulate the expression of CYP2E1 in cyclophosphamide-induced hepatotoxicity (Sheweita et al., 2016[76]).

The supplementation of 400 mg/kg of *P. minus* essential oil in PmEO400CP group up-regulates the CYP2E1 expression compared to control and cisplatin group in rat's liver. This might be due to the presence of aldehydes and alcohols in *P. minus* essential oil at high concentration in PmEO400CP which enhanced the CYP2E1 activity in the liver. Acetone, aldehyde and alcohol were cytochrome P450 isoenzyme inducers especially CYP2E1 (Ahmed Laskar and Younus, 2019[4]; Sánchez-Catalán et al., 2008[72]). The current findings is concurrent with Lu and Cederbaum (2006[54]) who demonstrated the combination of cisplatin and acetone increases the CYP2E1 activity two to four-fold as compared to cisplatin group alone. ROS produced by CYP2E1 together with the presence of aldehyde and alcohol might promote the formation of hydroxyl radicals and these radicals induce toxicity by interrupting the cell membrane through lipid peroxidation and the formation of MDA. In addition, Quintanilha and colleagues (2017[66]) also stated that CYP2E1-dependent oxidative stress can be synergized together with the cisplatin to potentiate further toxicity and oxidative damage. 

ROS can affect vital cell components especially lipid, proteins and DNA and cause cell damage (Krishnamurthy and Wadhwani, 2012[51]). Increased level of MDA and reduction in antioxidant status (GSH, GPx, SOD, and catalase) were observed in the cisplatin treated rats and this is in agreement with previous studies, i.e. Bishr and colleagues (2018[13]) as well as Ahmadipour and colleagues (2015[3]). GSH plays an important role in the detoxification of cisplatin and cell death occurs when GSH is markedly exhausted (Ince et al., 2014[41]). Reduced level of GPx, SOD and catalase in the cisplatin group might be due to higher consumption of these antioxidants due to enhanced oxidative stress in the liver (Kheiripour et al., 2016[48]). Supplementation with 100 mg/kg of *P. minus* essential oil in cisplatin-treated rats caused a significant decrease in MDA, protein carbonyl, 8-OHdG level and increased the antioxidant status implicating the protective effects of 100 mg/kg *P. minus* essential oil against cisplatin-induced hepatic oxidative damage. This finding is in agreement with previous studies which indicated that essential oil from *Foeniculum vulgare* decreased the hepatic MDA and increased the level of GSH, GPx, SOD and catalase activity in settings of cyclophosphamide-induced genotoxicity (Tripathi et al., 2013[80]). In addition,* Achillea wilhelmsii* essential oil lowered the protein carbonyl level in acetaminophen-induced liver injury (Dadkhah et al., 2015[26]). The activity of *P. minus* essential oil against oxidative stress might be due to the presence of ɑ-caryophyllene and β-caryophyllene which has been previously demonstrated to act as superoxide and hydroxyl radical scavenger by previous researchers (Hana et al., 2014[37]; Calleja et al., 2013[17]). Cyclolongifolene oxide is the main sesquiterpene found in *P. minus* essential oil and it was also found in high abundance in *Heteroxenia ghardaqensis *essential oil (Farrag et al., 2019[33]; Verma et al., 2011[83]). *H. ghardaqensis *essential oil showed similarly high antioxidant activities and protected against DNA damage in rats (Farrag et al., 2019[33]). Therefore, the reduction of 8-OHdG might be due to the presence of cyclolongifolene oxide. 

The supplementation of 200 mg/kg and 400 mg/kg of *P. minus* essential oil in PmEO200CP and PmEO400CP group showed no significant difference in MDA, protein carbonyl, 8-OHdG and antioxidant status compared to cisplatin group. Previous study demonstrated that essential oil could also cause oxidative stress. The percentage of aldehyde in *P. minus* essential oil is 52.47 %. Therefore, the higher dose will deliver more aldehyde to the rats. Therefore the concentration of essential oil must be chosen carefully. This finding is parallel with Lu and Cederbaum (2006[54]) who demonstrate the increase of MDA, AST and ALT level in rats treated with cisplatin and acetone. Aldehyde and acetone are much alike in many of their reactions owing to the presence of carbonyl functional group (Ouellette and Rawn, 2014[61]). Therefore, a high concentration of aldehyde that is present in a high concentration of *P. minus* essential oil combined with cisplatin could be the possible reasons for the changes in the antioxidant status seen in the PmEO400CP group. High concentration of aldehydes in combination with cisplatin might produce high toxic free radicals which cause oxidative stress (Ahmed Laskar and Younus, 2019[4]). 

TNF-α, IL-1 (α and β), IL-6 and IL-10 play an important role in regulating the interaction between inflammatory, oxidative stress and apoptotic pathways in cisplatin-induced hepatotoxicity (Shaw et al., 2011[75]; Tadagavadi and Reeves, 2010[78]). Cisplatin initiates inflammatory processes by promoting TNF-α production in residents and infiltrating immune cells (Damião et al., 2013[27]). TNF-α is also secreted from activated Kupffer cells which later worsens the oxidative stress and inflammatory response in hepatocytes (Augustyniak et al., 2005[8]). In this study, cisplatin produces inflammatory responses in hepatic tissues which was observed through the elevation in the level of pro-inflammatory cytokines (TNF-α, IL-1α, IL-1β, IL-6) and decreased level of anti-inflammatory cytokine (IL-10). These findings are in line with Rehman and colleagues (2014[68]) who demonstrated that cisplatin also caused elevation in level of TNF-α, IL-1β and IL-6 in liver. The present findings demonstrated that *P. minus* essential oil at the dose of 100 mg/kg reduced the inflammatory response in liver induced by cisplatin. The supplementation of PmEO100CP lowered the level of TNF-α, IL-1α, IL-1β and IL-6 and increased the level of IL-10. The reversal of this inflammatory response might be due to the presence of sesquiterpenes in *P. minus* essential oil that can reduce the complement activation, thus decreasing adhesion of inflammatory cells to the endothelium that results in inflammatory reaction (Chen et al., 2017[20]; Rogerio et al., 2009[69]). In addition, sesquiterpenes also may inhibit metabolism of arachidonic acid which acts as inducer for pro-inflammatory activation (Hana et al., 2014[37]).

*P. minus* essential oil also consists of α-caryophyllene and according to Fernandes and colleagues (2007[34]), α-caryophyllene was found to inhibit TNF-α signaling pathway and suppress the IL-1β and IL-6 generation in carrageenan-induced rats. Besides, β-caryophyllene also exhibited the anti-inflammatory effect against cisplatin-induced nephropathy in a murine model. In addition, α-caryophyllene and β-caryophyllene were found to inhibit the lipopolysaccharide (LPS)-induced NF-kB activation and neutrophil migration in animal models of inflammation (Medeiros et al., 2007[57]). Valencene, and seychellene were found to be among sesquiterpenes found in *P. minus* essential oil. Tsoyi and colleagues (2011[82]) proved that valencene, which is the main active compound in *Cyperus rotundus,* induced expression of Heme oxygenase-1 (HO-1) responsible for anti-inflammatory reaction in mice. On the other hand, seychellene which was found in *Pogostemon cablin* also down-regulated the expression of TNF-α, IL-1β and IL-6 in LPS-induced RAW264.7 cells (Su et al., 2012[77]). Bisabolene, α-bergamotene and β-farnesene commonly found in *P. minus* essential oil which possessed anti-inflammatory effects against adjuvant-induced arthritis in rats also showed an effective result in decreasing oxidative stress in the liver (Castro Ghizoni et al., 2017[18]).

Meanwhile, the supplementation of 200 mg/kg and 400 mg/kg *P. minus* essential oil showed significantly higher level of TNF-α, IL1-α, IL-1β, IL-6 and significantly lower level of IL-10 compared to control group and also no differences compared to cisplatin group, which revealed that higher concentration of *P. minus* essential oil might not protect the liver from inflammation process. The interaction of cisplatin and aldehyde through the activation of CYP2E1 will accelerate the production ROS which activate the TNF-a signaling pathways and the release of inflammatory cytokine such as IL-1a, IL-1B and IL-6 (Vyas et al., 2014[84]). This finding is probably due to high concentration long-chain aldehyde such as decanal and dodecanal (dodecyl aldehyde) at 10.4 % and 39.4 %, respectively as well as the presence of cisplatin. 

Various studies have demonstrated that cisplatin induces apoptosis in the hepatic cells by interfering with DNA repair mechanisms, causing DNA damage and subsequently inducing apoptosis (Omar et al., 2016[60]). Cisplatin-induced apoptosis in hepatic tissue is initiated by cellular oxidative stress through the generation of ROS such as superoxide anion and hydroxyl radicals. ROS activities then modulate the activity of p53, Bax and Bcl-2 (Omar et al., 2016[60]) as well as the release of cytochrome c from mitochondria into the cytosol (Dasari and Tchounwou, 2014[28]). This will activate caspase 8, caspase 9 (in the presence of Apaf-1) and caspase 3 (Azuma et al., 2003[9]; Omar et al., 2016[60]). The present study demonstrated higher gene expression of p53, Bax, caspase 8, Apaf-1, caspase 9 and caspase 3 in cisplatin-induced hepatotoxicity which is in line with previous studies (Khan et al., 2011[46]). 

Supplementation of *P. minus* essential oil at the dose of 100 mg/kg lowered the gene expression of p53, Bax, cytochrome c, caspase 8, Apaf-1, caspase 9, caspase 3 and higher the Bcl-2 compared to the cisplatin group. This might be due to the antioxidant and anti-apoptotic properties of *P. minus *essential oil as suggested by Dasari and Tchounwou (2014[28]). α-Caryophyllene and β-caryophyllene are sesquiterpenes that were reported to have anti-oxidant property which reduced the lipid peroxidation, increased the antioxidant status, protected against the DNA damage (Sheweita et al., 2016[76]) and ameliorated apoptosis (Mahmoud et al., 2014[56]). In addition, β-caryophyllene reduced caspase 3 activity in cisplatin-induced cell death model *in vitro* (Horváth et al., 2012[39]). Meanwhile, valencene and seychellene are also among sesquiterpene compounds in *P. minus* essential oil. Valencene was believed to efficiently enhance Bcl-2 and decrease caspase 3 expression in 3-morpholinosydnonimine (SIN-1)-induced apoptosis (Hemanth Kumar et al., 2013[38]). Seychellene is one of the main compound in *P. cablin*, and has been suggested to prevent the release of cytochrome c into cytosol in human neuroglioma cell line induced by hydrogen peroxide (H_2_O_2_) (Kim et al., 2010[49]). Therefore, at the dose of the 100 mg/kg *P. minus* essential oil, sesquiterpenes compound was suggested to play the main role in protecting apoptosis.

The combination of cisplatin with higher dose of *P. minus* essential oil increases the p53, caspase 8, Apaf-1, caspase 9 and caspase 3 expression in rat's liver. The production of higher level of ROS in PmEO400CP group activates the activity of p53 protein which further disrupts the mitochondrial membrane permeabilization by down-regulating the pro-survival protein such as Bcl-2 and upregulating pro-apoptotic protein such as Bax, caspase 8, Apaf-1, caspase 9 and caspase 3 (Redza-Dutordoir and Averill-Bates, 2016[67]). This observation supports Pavithra and colleagues (2018[65]) who demonstrated that the high concentration of *Pamburus missionis* essential oil exerts the activity of apoptosis in A431 and HaCaT cells. This was due to the increased ROS level in the high concentration of *P. missionis* essential oil. In addition, *P. missionis* essential oil also contains aldehyde and alcohols which contribute to the increase of the ROS level in the treated cells (Pavithra et al., 2018[65]).

β-Caryophyllene is a FDA-approved food additive that contains many medicinal properties such as antioxidant (Sharma et al., 2016[74]), anti-inflammatory (Bento et al., 2011[12]), antimicrobial (Donati et al., 2015[30]), anti-apoptosis (Horváth et al., 2012[39]) and chemopreventive effects (Bridgeman and Abazia, 2017[15]). The protective effects of β-caryophyllene on liver have been confirmed in a liver fibrosis model induced by surgical ligation of the common bile duct (Mahmoud et al., 2014[56]) and in carbon tetrachloride-induced liver fibrosis (Calleja et al., 2013[17]) whereby it normalized liver transaminase and bilirubin level. Furthermore, when combined with silymarin, it was also found to ameliorate ketoprofen-induced hepatotoxicity in rats (Kelany and Abdallah, 2016[45]). In the present study, the supplementation of β-caryophyllene significantly lowered the level of liver enzyme and bilirubin. Therefore, this explains its role as a potential hepatoprotective agent. The present finding supports (Horvath et al., 2012[39]) where in cisplatin-induced nephrotoxicity, β-caryophyllene was found to reduce the antioxidant status, inflammatory markers (TNF-α and IL-1β) and apoptosis marker (caspase 3).

In the present study, the concentration of β-caryophyllene supplied via essential oil in PmEO100CP group is not as much as that administered in the β-caryophyllene group, however both groups demonstrated comparable and similar antioxidant, anti-inflammatory and anti-apoptotic effects. These effects shown by the PmEO100CP group could be due to the presence of other compounds in *P. minus* essential oil that might work synergistically. 

Meanwhile, the supplementation of *P. minus* essential oil at a higher concentration especially in PmEO400CP showed contrary results with the PmEO100CP group. This finding suggested that at higher concentration of *P. minus* essential oil might not protect the liver from oxidative stress, inflammation and apoptosis in cisplatin-induced hepatotoxicity. However, these negative observations were not significantly in PmEO400 alone. The expression of CYP2E1 was also found significantly higher in the PmEO400CP group. These findings suggested that the compounds present at a higher dose in *P. minus* essential oil might interact with cisplatin to increase CYP2E1 which then augments oxidative stress, inflammation and apoptosis.

The present study showed that *P. minus* essential oil contained high concentration of long-chain aldehyde (52.47 %). A study done by Lu and Cederbaum (2006[54]) showed a combination of acetone and cisplatin in increasing the production of ROS which then potentiates the cellular oxidative stress and reduction in GSH levels in the* in vitro* model. Higher concentration of aldehyde that is present in the high concentration of *P. minus* essential oil could be the possible reason for the pro-oxidant, pro-inflammatory and pro-apoptotic effects seen in PmEO400CP group.

**Figure 5:**
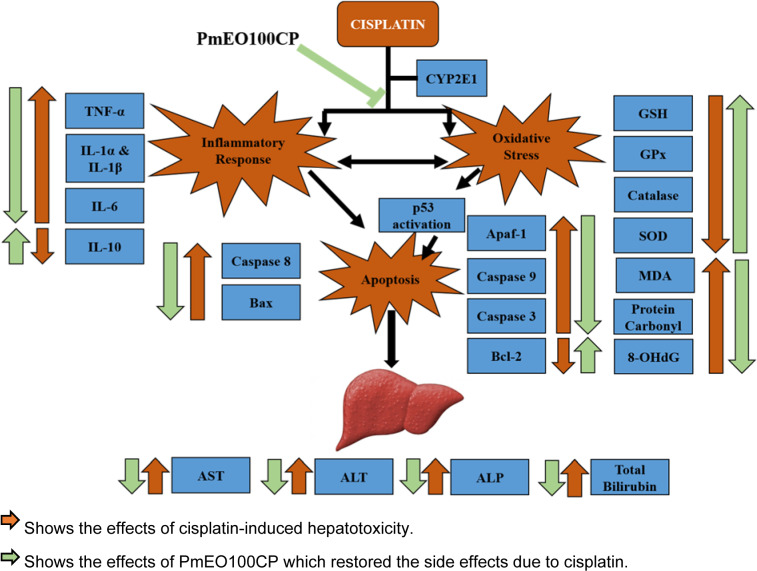
Overview of the effects of 100 mg/kg of *P. minus* essential oil against cisplatin-induced hepatotoxicity. At a dose of 100 mg/kg of *P. minus* essential oil able to ameliorate the effects of cisplatin-induced hepatotoxicity in rats by improving the antioxidant status and reducing the oxidative stress. In addition, it also restored the inflammatory response and apoptosis markers. As a consequence, 100 mg/kg of *P. minus* essential oil might be able to prevent liver injury in cisplatin-induced hepatotoxicity.

Figure 5(Fig. 5) shows the overview effect of 100 mg/kg *P. minus* essential oil in cisplatin-induced hepatotoxicity.

## Conclusion

In conclusion, our results showed that *P. minus* essential oil at low dose (100 mg/kg) might protect against cisplatin-induced hepatotoxicity via inhibition of oxidative stress, inflammation and apoptosis. However, at higher concentration, *P. minus* essential oil showed pro-oxidant, pro-inflammatory and pro-apoptotic effects and therefore it is important to be cautious for the supplementation at higher dose to prevent any detrimental effects.

## Notes

Norhashima Abd Rashid, Farida Hussan, Asmah Hamid, Nurul Raudzah Adib Ridzuan, Syarifah Aisyah Syed Abd Halim, Nahdia Afiifah Abdul Jalil, Nor Haliza Mohamad Najib and Seong Lin Teoh contributed equally to this work. 

## Acknowledgements

This study was funded by FRGS/2/2014/SKK01/UKM/02/1 from the Ministry of Higher Education of Malaysia. The Ph. D. fees and allowance of the main author was funded by the Ministry of Higher Education of Malaysia. The authors also thank the staff of the Faculty of Health Sciences and Department of Anatomy, PPUKM for their technical advice.

## Conflict of interest

The authors have declared that no conflict of interest exists.

## Supplementary Material

Supplementary information

## Figures and Tables

**Table 1:**
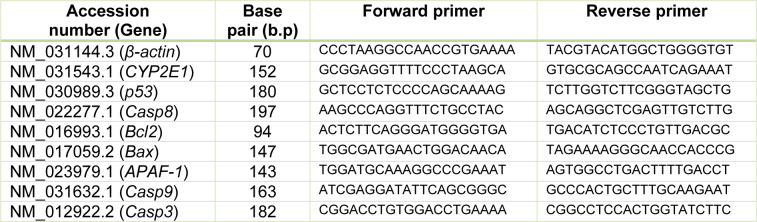
Forward and reverse primer sequence used in qPCR analysis

**Table 2:**
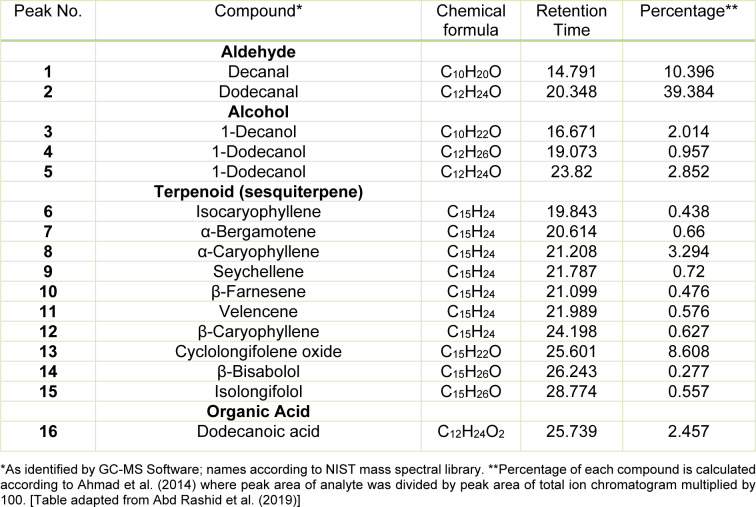
Major volatile compound identified in *P. minus* essential oil

**Table 3:**
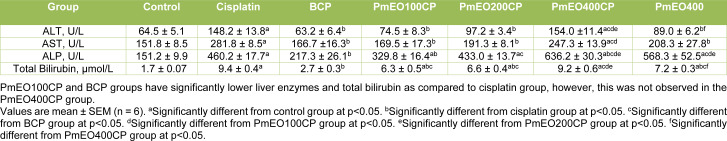
Effect of *P. minus* essential oil on liver function in cisplatin-induced hepatotoxicity rats

**Table 4:**
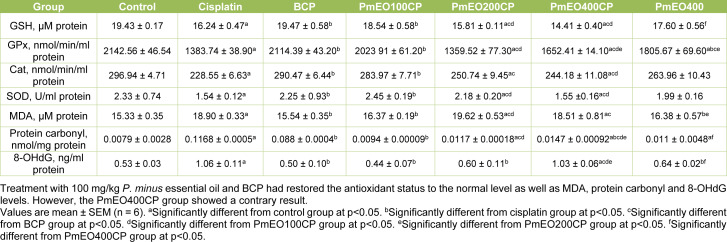
Effect of *P. minus* essential oil on antioxidant and oxidative stress biomarkers in the liver of cisplatin-induced hepatotoxicity rats
