# Supplementary material for: Polygonum minus essential oil modulates cisplatin-induced hepatotoxicity through inflammatory and apoptotic pathways
Source: EXCLI J. 2020 Sep 9;19:1246–65. doi: 10.17179/excli2020-2355 (PMC7590832; doi:10.17179/excli2020-2355)
Supplement: Supplementary information [file EXCLI-19-1246-s-001.pdf]

EXCLI Journal 2020;19:1246-1265 – ISSN 1611-2156
Received: May 02, 2020, accepted: September 01, 2020, published: September 09, 2020

Supplementary information to:

POLYGONUM MINUS ESSENTIAL OIL MODULATES CISPLATIN-INDUCED HEPATOTOXICITY THROUGH INFLAMMATORY AND APOPTOTIC PATHWAYS

Norhashima Abd Rashid[1¶], Farida Hussan[2¶], Asmah Hamid[3¶], Nurul Raudzah Adib Ridzuan[4¶], Syarifah Aisyah Syed Abd Halim[5¶], Nahdia Afiifah Abdul Jalil[5¶], Nor Haliza Mohamad Najib[5¶], Seong Lin Teoh[5¶], Siti Balkis Budin[1*]

[1] Center for Diagnostic, Therapeutic and Investigative Studies, Faculty of Health Sciences, Universiti Kebangsaan Malaysia, Kuala Lumpur, Malaysia
[2] Human Biology Department, School of Medicine, International Medical University, Bukit Jalil, Kuala Lumpur, Malaysia
[3] Centre for Toxicology and Health Risk Studies, Faculty of Health Sciences, Universiti Kebangsaan Malaysia, Kuala Lumpur, Malaysia
[4] Department of Anatomy, Faculty of Medicine, Universiti Teknologi MARA, Selangor, Malaysia
[5] Department of Anatomy, Faculty of Medicine, Universiti Kebangsaan Malaysia Medical Centre, Kuala Lumpur, Malaysia

¶ These authors contributed equally to this work.

* **Corresponding author:** Siti Balkis Budin, Center for Diagnostic, Therapeutic and Investigative Studies, Faculty of Health Sciences, Universiti Kebangsaan Malaysia, Jalan Raja Muda Abdul Aziz, 50300 Kuala Lumpur, Malaysia. E-mail: balkis@ukm.edu.my

http://dx.doi.org/10.17179/excli2020-2355

This is an Open Access article distributed under the terms of the Creative Commons Attribution License (http://creativecommons.org/licenses/by/4.0/).

EXCLI Journal 2020;19:1246-1265 – ISSN 1611-2156
Received: May 02, 2020, accepted: September 01, 2020, published: September 09, 2020

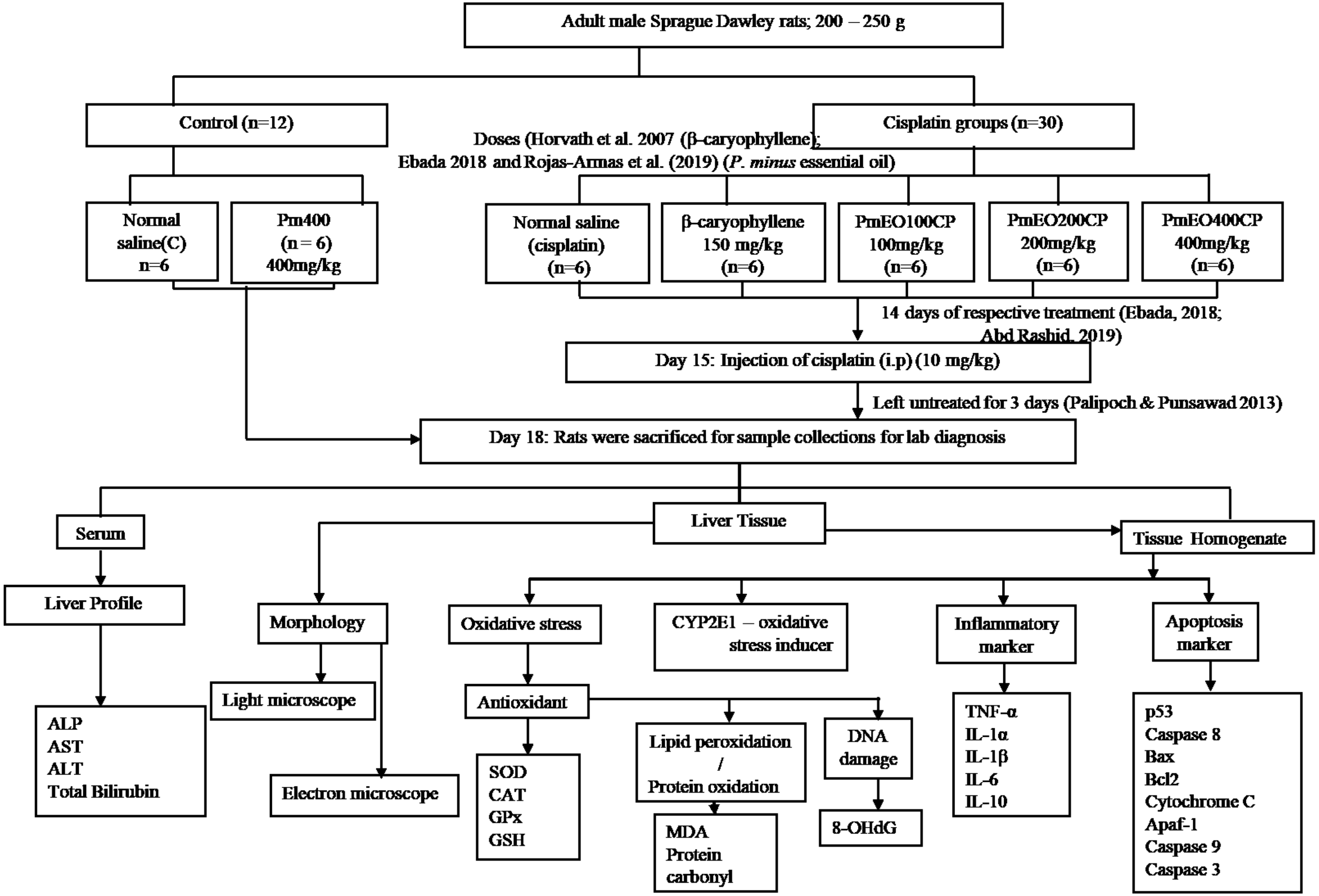

Supplementary Figure 1: Flowchart of the effect of *P. minus* essential oil against cisplatin-induced hepatotoxicity

EXCLI Journal 2020;19:1246-1265 – ISSN 1611-2156
Received: May 02, 2020, accepted: September 01, 2020, published: September 09, 2020

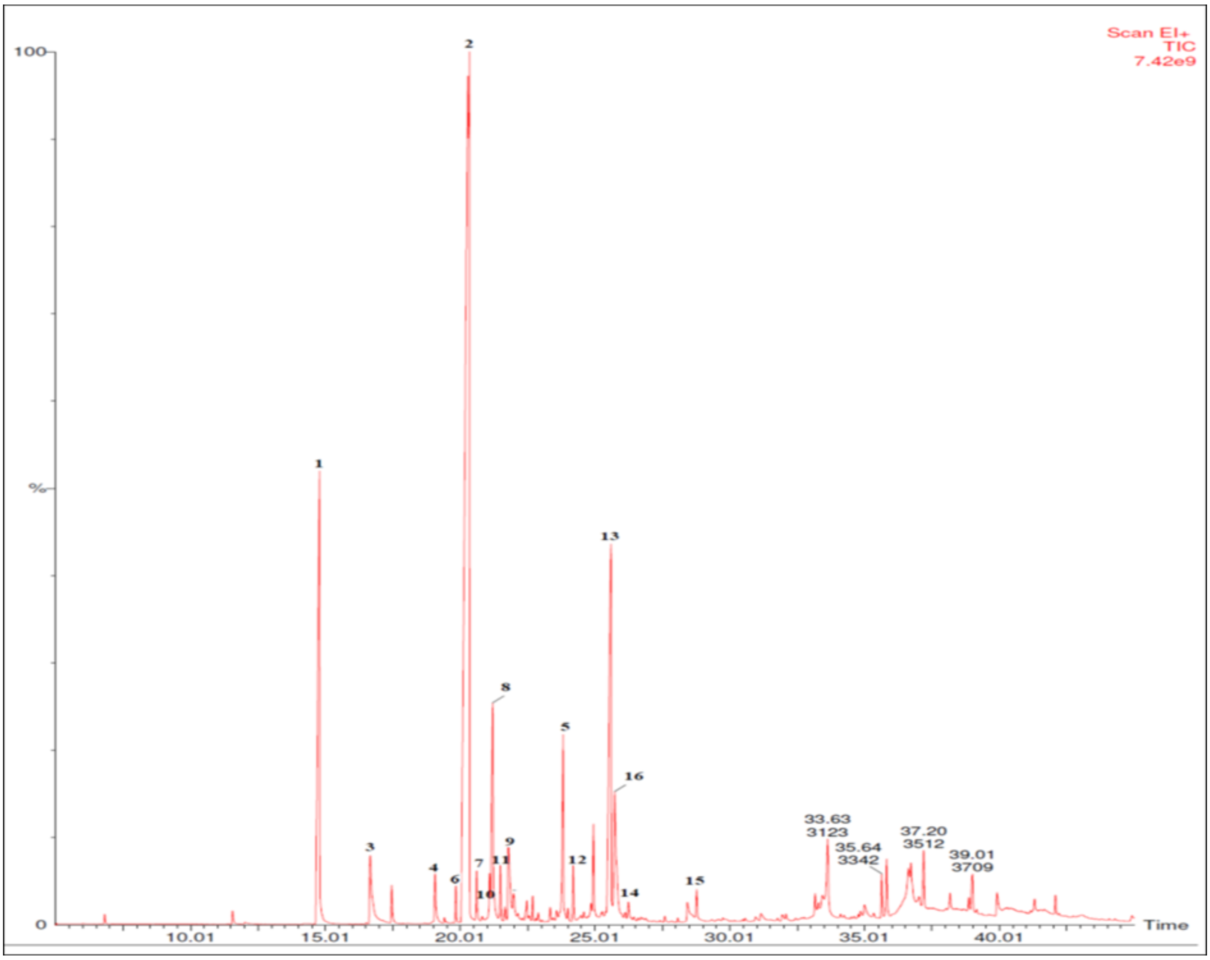

Supplementary Figure 2: MS chromatogram of *P. minus* essential oil

EXCLI Journal 2020;19:1246-1265 – ISSN 1611-2156
Received: May 02, 2020, accepted: September 01, 2020, published: September 09, 2020

Supplementary Table 1: The mean and SEM of the effect of *P. minus* essential oil on CYP2E1 gene expression level in the liver of cisplatin-induced hepatotoxicity rats

Groups	Relative mRNA expression
	**Mean ± SEM**
**Control (Normal Saline)**	1.00 ± 0.10
**Cisplatin (10 mg/kg)**	1.88 ± 0.24[a]
**BCP (150 mg/kg)**	1.04 ± 0.11[b]
**PmEO100CP**<br>**(*P. minus* Essential Oil 100 mg/kg + Cisplatin 10 mg/kg)**	1.14 ± 0.07[b]
**PmEO200CP**<br>**(*P. minus* Essential Oil 200 mg/kg + Cisplatin 10 mg/kg)**	1.43 ± 0.10[b]
**PmEO400CP**<br>**(*P. minus* Essential Oil 400 mg/kg + Cisplatin 10 mg/kg)**	2.40 ± 0.34[abcde]
**PmEO400**<br>**(*P. minus* Essential Oil 400 mg/kg)**	1.74 ±0.06[acdf]

Values are mean ± SEM (n = 6).

[a]Significantly different from control group at $p<0.05$.

[b]Significantly different from cisplatin group $p<0.05$.

[c]Significantly different from BCP group at $p<0.05$.

[d]Significantly different from PmEO100CP group at $p<0.05$.

[e]Significantly different from PmEO200CP group at $p<0.05$.

[f]Significantly different from PmEO400CP group at $p<0.05$.

EXCLI Journal 2020;19:1246-1265 – ISSN 1611-2156
Received: May 02, 2020, accepted: September 01, 2020, published: September 09, 2020

Supplementary Table 2: The mean and SEM of the effect of *P. minus* essential oil on inflammation markers in the liver of cisplatin-induced hepatotoxicity rats

Groups	TNF-α	IL-1α	IL-1β	IL-6	IL-10
	Mean ± SEM	Mean ± SEM	Mean ± SEM	Mean ± SEM	Mean ± SEM
**Control (Normal Saline)**	3.14 ± 0.30	138.01 ± 4.6	563.17 ± 35.35	3661.51 ± 208.30	912.31 ± 65.04
**Cisplatin (10 mg/kg)**	7.20 ± 0.37[a]	357.22 ± 10.91[a]	915.30 ± 32.87[a]	5030.50 ± 199.94[a]	636.46 ± 18.20[a]
**BCP (150 mg/kg)**	4.47 ± 0.54[b]	151.82 ± 6.93[b]	676.74 ± 15.40[b]	3854.20 ± 136.29[b]	972.98 ± 59.44[b]
**PmEO100CP (*P. minus* Essential Oil 100 mg/kg + Cisplatin 10 mg/kg)**	4.01 ± 0.70[b]	194.10 ± 24.83[b]	591.46 ±74.60[b]	3390.46 ± 408.90[b]	878.60 ± 68.90[b]
**PmEO200CP (*P. minus* Essential Oil 200 mg/kg + Cisplatin 10 mg/kg)**	6.30 ± 0.37[a]	298.24 ± 22.45[acd]	865.25 ± 60.06[ad]	6001.87 ± 197.33[acd]	576.75 ± 54.76[acd]
**PmEO400CP (*P. minus* Essential Oil 400 mg/kg + Cisplatin 10 mg/kg)**	9.42 ± 1.08[acde]	304.98 ± 26.91[acd]	828.90 ± 56.13[ad]	4849.26 ± 108.71[ade]	500.82 ± 42.48[acd]
**PmEO400 (*P. minus* Essential Oil 400 mg/kg)**	3.35 ± 0.48[bef]	191.96 ± 11.81[bef]	553.41 ± 48.05[bef]	3431.50 ± 291.75[bef]	504.68 ± 34.27[acd]

Values are mean ± SEM (n=6).

[a]Significantly different from control group at p<0.05.

[b]Significantly different from cisplatin group at p<0.05.

[c]Significantly different from BCP group at p<0.05.

[d]Significantly different from PmEO100CP group at p<0.05.

[e]Significantly different from PmEO200CP group at p<0.05.

[f]Significantly different from PmEO400CP group at p<0.05.

Received: May 02, 2020, accepted: September 01, 2020, published: September 09, 2020

Supplementary Table 3: The mean and SEM of the effect of *P. minus* essential oil on apoptosis gene expression in the liver of cisplatin-induced hepatotoxicity rats

Groups	Relative mRNA expression						
	p53	Caspase 8	Bax	Apaf-1	Caspase 9	Caspase 3	Bcl-2
	Mean ± SEM	Mean ± SEM	Mean ± SEM	Mean ± SEM	Mean ± SEM	Mean ± SEM	Mean ± SEM
**Control (Normal Saline)**	1.00 ± 0.02	1.00 ± 0.00	1.00 ± 0.00	1.00 ± 0.00	1.00 ± 0.00	1.00 ± 0.00	1.00 ± 0.10
**Cisplatin (10 mg/kg)**	2.00 ± 0.02[a]	2.51 ± 0.00[a]	1.67 ±0.00[a]	2.80 ± 0.00[a]	4.94 ± 0.00[a]	6.26 ± 0.06[a]	0.25 ± 0.00[a]
**BCP (150 mg/kg)**	0.64 ± 0.01[b]	1.19 ± 0.01[b]	1.31 ± 0.00[b]	1.31 ±0.00[b]	1.11 ± 0.00[b]	2.98 ± 0.09[b]	0.89 ± 0.00[b]
**PmEO100CP (*P. minus* Essential Oil 100 mg/kg + Cisplatin 10 mg/kg)**	0.59 ± 0.01[b]	1.36 ± 0.00[b]	1.09 ± 0.00[b]	1.32 ± 0.00[b]	1.22 ± 0.00[b]	2.50 ± 0.05[b]	0.85 ± 0.00[b]
**PmEO200CP (*P. minus* Essential Oil 200 mg/kg + Cisplatin 10 mg/kg)**	0.82 ± 0.01[b]	1.84 ± 0.02	1.86 ± 0.00[acd]	2.00 ± 0.01	2.27 ± 0.00	5.25 ± 0.04[ad]	0.34 ± 0.00[a]
**PmEO400CP (*P. minus* Essential Oil 400 mg/kg + Cisplatin 10 mg/kg)**	2.96 ± 0.04[acde]	2.42 ± 0.02[ac]	1.87 ± 0.00[acd]	4.56 ± 0.04[abcde]	6.07 ± 0.02[acde]	7.99 ± 0.07[acde]	0.05 ± 0.00[acd]
**PmEO400 (*P. minus* Essential Oil 400 mg/kg)**	0.80 ± 0.07[bf]	1.60 ± 0.01	1.22 ± 0.00[ef]	2.55 ± 0.01[bef]	0.96 ± 0.00[bf]	1.01 ± 0.03[bef]	0.39 ± 0.00[a]

Values are mean ± SEM (n = 6).

[a] Significantly different from control group at p<0.05.

[b] Significantly different from cisplatin group at p<0.05.

[c] Significantly different from BCP group at p<0.05.

[d] Significantly different from PmEO100CP group at p<0.05.

[e] Significantly different from PmEO200CP group at p<0.05.

[f] Significantly different from PmEO400CP group at p<0.05.

EXCLI Journal 2020;19:1246-1265 – ISSN 1611-2156
Received: May 02, 2020, accepted: September 01, 2020, published: September 09, 2020

Supplementary Table 4: The mean and SEM of the effect of *P. minus* essential oil on cytochrome c protein expression in the liver of cisplatin-induced hepatotoxicity rats

Groups	Expression Level (Fold Change)
	Mean ± SEM
**Control (Normal Saline)**	1.00 ± 0.05
**Cisplatin (10 mg/kg)**	2.18 ± 0.01[a]
**BCP (150 mg/kg)**	1.18 ± 0.00[b]
**PmEO100CP (*P. minus* Essential Oil 100 mg/kg + Cisplatin 10 mg/kg)**	0.95 ± 0.02[b]
**PmEO200CP (*P. minus* Essential Oil 200 mg/kg + Cisplatin 10 mg/kg)**	1.86 ± 0.02[ad]
**PmEO400CP (*P. minus* Essential Oil 400 mg/kg + Cisplatin 10 mg/kg)**	2.31 ± 0.03[acd]
**PmEO400 (*P. minus* Essential Oil 400 mg/kg)**	1.68 ± 0.05[ad]

[a]Significantly different from control group at $p<0.05$.

[b]Significantly different from cisplatin group at $p<0.05$.

[c]Significantly different from BCP group at $p<0.05$.

[d]Significantly different from PmEO100CP group at $p<0.05$.
